# Effects of a peripherally acting µ-opioid receptor antagonist for the prevention of recurrent acute pancreatitis: study protocol for an investigator-initiated, randomized, placebo-controlled, double-blind clinical trial (PAMORA-RAP trial)

**DOI:** 10.1186/s13063-023-07287-z

**Published:** 2023-05-01

**Authors:** Mathias E. Cook, Cecilie S. Knoph, Camilla A. Fjelsted, Jens B. Frøkjær, Anders E. Bilgrau, Srdan Novovic, Maiken Thyregod Jørgensen, Michael B. Mortensen, Liv B. J. Nielsen, Amer Hadi, Mark Berner-Hansen, Wiktor Rutkowski, Miroslav Vujasinovic, Matthias Löhr, Asbjørn M. Drewes, Søren S. Olesen

**Affiliations:** 1 Department of Gastroenterology and Hepatology, Centre for Pancreatic Diseases and Mech-Sense, Aalborg University Hospital, Aalborg, Denmark; 2 Department of Clinical Medicine, Aalborg University, Aalborg, Denmark; 3 Department of Radiology, Aalborg University Hospital, Aalborg, Denmark; 4 Department of Mathematical Sciences, Aalborg University, Aalborg, Denmark; 5 Copenhagen University Hospital Hvidovre, Hvidovre, Denmark; 6 Department of Surgery, Odense Pancreas Centre (OPAC), HPB Section, Odense University Hospital, Odense, Denmark; 7 Digestive Disease Centre K, Bispebjerg Hospital, University of Copenhagen, Copenhagen, Denmark; 8 Department for Upper Abdominal Diseases, Karolinska University Hospital, Stockholm, Sweden

**Keywords:** Peripherally acting µ-opioid receptor antagonist, Recurrent acute pancreatitis, Randomized controlled trial, Naldemedine, Opioid, Magnetic resonance imaging

## Abstract

**Background:**

Acute and chronic pancreatitis constitute a continuum of inflammatory disease of the pancreas with an increasing incidence in most high-income countries. A subset of patients with a history of pancreatitis suffer from recurrence of acute pancreatitis attacks, which accelerate disease progression towards end-stage chronic pancreatitis with loss of exocrine and endocrine function. There is currently no available prophylactic treatment for recurrent acute pancreatitis apart from removing risk factors, which is not always possible. Pain is the primary symptom of acute pancreatitis, which induces the endogenous release of opioids. This may further be potentiated by opioid administration for pain management. Increased exposure to opioids leads to potentially harmful effects on the gastrointestinal tract, including, e.g. increased sphincter tones and decreased fluid secretion, which may impair pancreatic ductal clearance and elevate the risk for new pancreatitis attacks and accelerate disease progression. Peripherally acting µ-opioid receptor antagonists (PAMORAs) have been developed to counteract the adverse effects of opioids on the gastrointestinal tract. We hypothesize that the PAMORA naldemedine will reduce the risk of new pancreatitis attacks in patients with recurrent acute pancreatitis and hence decelerate disease progression.

**Methods:**

The study is a double-blind, randomized controlled trial with allocation of patients to either 0.2 mg naldemedine daily or matching placebo for 12 months. A total of 120 outpatients will be enrolled from five specialist centres in Denmark and Sweden. The main inclusion criteria is a history of recurrent acute pancreatitis (minimum of two confirmed pancreatitis attacks). The primary endpoint is time to acute pancreatitis recurrence after randomization. Secondary outcomes include changes in quality of life, gastrointestinal symptom scores, new-onset diabetes, exocrine pancreatic insufficiency, disease severity, health care utilization, adherence to treatment, and frequency of adverse events. Exploratory outcomes are included for mechanistic linkage and include the progression of chronic pancreatitis-related findings on magnetic resonance imaging (MRI) and changes in circulating blood markers of inflammation and fibrosis.

**Discussion:**

This study investigates if naldemedine can change the natural course of pancreatitis in patients with recurrent acute pancreatitis and improve patient outcomes.

**Trial registration:**

EudraCT no. 2021–000069-34. ClinicalTrials.gov NCT04966559. Registered on July 8, 2021.

## Administrative information

Note: the numbers in curly brackets in this protocol refer to SPIRIT checklist item numbers. The order of the items has been modified to group similar items (see http://www.equator-network.org/reporting-guidelines/spirit-2013-statement-defining-standard-protocol-items-for-clinical-trials/).

**Table:** 

Title {1}	Effects of a peripherally acting µ-opioid receptor antagonist for the prevention of recurrent acute pancreatitis: study protocol for an investigator-initiated, randomized, placebo-controlled, double-blind clinical trial. (PAMORA-RAP trial).
Trial registration {2a and 2b}.	EudraCT no. 2021–000069-34 Clinicaltrials.gov NCT04966559 registered July 8, 2021, https://clinicaltrials.gov/ct2/show/NCT04966559
Protocol version {3}	22.11.2021 Version 1.22
Funding {4}	The study is funded as part of an unrestricted grant by the Novo Nordisk Foundation. Shionogi BV also supports the study through a supply of study drugs.
Author details {5a}	^1^ Centre for Pancreatic Diseases and Mech-Sense, Department of Gastroenterology and Hepatology, Aalborg University Hospital, Aalborg, Denmark ^2^ Department of Clinical Medicine, Aalborg University, Aalborg, Denmark ^3^ Department of Radiology, Aalborg University Hospital, Aalborg, Denmark ^4^ Department of Mathematical Sciences, Aalborg University, Aalborg, Denmark ^5^ Gastrounit, Copenhagen University Hospital Hvidovre, Hvidovre, Denmark ^6^ Odense Pancreas Centre (OPAC), HPB Section, Department of Surgery, Odense University Hospital, Odense, Denmark ^7^ Digestive Disease Centre K, Bispebjerg Hospital, University of Copenhagen, Copenhagen, Denmark ^8^ Department for Upper Abdominal Diseases, Karolinska University Hospital, Stockholm, Sweden
Name and contact information for the trial sponsor {5b}	Asbjørn Mohr Drewes, Professor, MD, PhD, DMSc Centre for Pancreatic Diseases and Mech-Sense Department of Gastroenterology & Hepatology Aalborg University Hospital 9000 Aalborg, Denmark Telephone: + 45 97 66 35 62 E-mail: amd@rn.dk
Role of sponsor {5c}	The PAMORA-RAP study is an investigator-initiated trial. The financial supporters have no influence on study design, data collection, or publication.

## Introduction

### Background and rationale {6a}

Acute and chronic pancreatitis constitute a continuum of fibro-inflammatory disease processes of the pancreas. Patients are often characterized by one or more predisposing risk factors (e.g. smoking, excessive alcohol consumption, gallstones, and genetic predisposition) [1]. If the predisposing risk factors are not modified or removed, there is a high risk of disease recurrence with ensuing tissue damage and disease progression towards end-stage chronic pancreatitis. Hence, approximately 10% of patients who have had a single attack of acute pancreatitis will progress to chronic pancreatitis, while the progression rate increases to 20–30% in patients with recurrent acute pancreatitis [2]. Especially in patients with alcoholic pathophysiology in their first-time acute pancreatitis attack, the progression rate increases significantly compared to other aetiologies [3].

In many cases, recurrent pancreatitis can be related to continuing toxic exposure to smoking and alcohol or unrecognized or inadequately treated biliary stones [4]. However, in 10–15% of cases, the aetiology remains unexplained despite a thorough diagnostic workup [5]. Currently, no prophylactic treatment is available for these patients, who have a high risk of recurrent episodes of pancreatitis. Also, many patients with chronic pancreatitis experience acute flares of pancreatic inflammation (i.e. acute pancreatitis) typically characterized by pain exacerbation that is often managed by opioid prescription. This condition has been termed acute on chronic pancreatitis and contributes to reduced quality of life, increased hospitalization rates, and missed workdays [6]. The current consensus definition of recurrent acute pancreatitis is two or more acute pancreatitis episodes with a complete resolution between the attacks [5]. In the present study, we use the term recurrent acute pancreatitis to include recurring flares of confirmed pancreatitis in patients at various stages on the pancreatitis continuum, which is in line with other ongoing studies [7, 8].

Pain is the cardinal symptom of acute pancreatitis and mediates the release of endogenous opioids, which are further potentiated by exogenously administered opioids used for pain management. Importantly, a large proportion of patients with recurrent and chronic pancreatitis also suffer from abdominal pain between flares of pancreatitis and may be exposed to extended periods of opioid treatment [1]. Opioid exposure is associated with several harmful effects on the gastrointestinal organs that may worsen the disease course of pancreatitis [9]. Most importantly, opioids directly affect the pancreas where they decrease fluid secretion from ductal cells and increase the tonus of the sphincter of Oddi, which in combination may lead to impaired pancreatic ductal clearance of activated pancreatic enzymes and other harmful substances [10]. This promotes intrapancreatic activation of trypsinogen and may initiate a new inflammatory attack (i.e. recurrent pancreatitis) [11]. Exogenously administered opioids have also been shown to have an immunosuppressive effect on both the innate and adaptive immune systems in animal studies. However, these effects have not been sufficiently investigated in human studies [12]. Additionally, increased levels of endogenous and exogenous opioids potentially decrease small intestine motility, which increases the risk of small intestine bacterial overgrowth and impaired intestinal integrity [13, 14]. Together these adverse effects can lead to the translocation of bacteria to the systemic circulation and further potentiate the severity of pancreatitis [15]. Finally, opioid exposure is associated with an excess risk of constipation [13]. In severe cases, this may lead to worsening of abdominal pain and hospitalization due to suspicion of a new pancreatitis attack. Altogether, opioids have several harmful effects on the pancreatic gland and gastrointestinal tract and thus may impact the clinical course of recurrent pancreatitis.

**Fig. 1:**
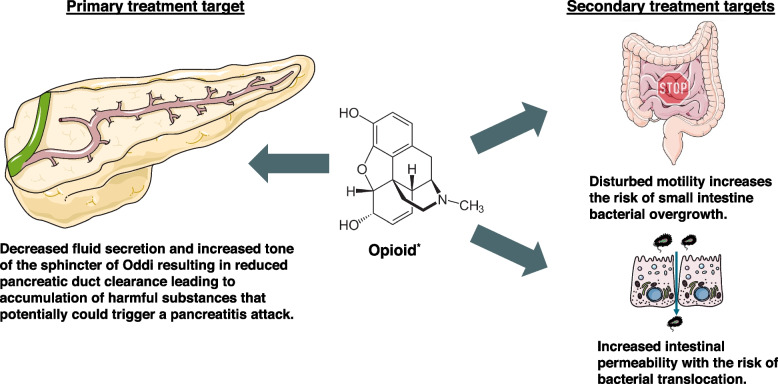
Summary of the harmful effects of endo- and exogenous opioids on the pancreatic gland and gastrointestinal tract in patients with recurrent pancreatitis. Primary and secondary treatment targets for PAMORA are illustrated. *Opioid represented as a morphine molecule

Peripherally acting µ-opioid receptor antagonists (PAMORAs) have been developed to treat opioid-induced bowel dysfunction [16]. As outlined above, the tone in the sphincter of Oddi and pancreatic secretion is partly controlled by endogenous opioids, and during pain attacks, these are upregulated. Therefore, treatment with PAMORAs may counteract the abovementioned adverse effects of endogenous opioids on the pancreatic gland and gastrointestinal tract. This effect may be even more pronounced if exogenous opioids are used in pain management. PAMORAs have, however, never been investigated for this indication. Potentially, the administration of PAMORAs in patients with recurrent pancreatitis is expected to increase pancreatic duct flow [13] and facilitate the clearance of putative pathogenic substances, including intrapancreatic prematurely activated trypsinogen. They may also antagonize the potentially harmful effects of opioids on intestinal integrity and the immune system. The harmful effects of opioids on the pancreatic gland and gastrointestinal organs are illustrated in Fig. 1, along with the treatment targets of PAMORA in relation to recurrent acute pancreatitis.

### Objectives {7}

The primary objective of this trial is to investigate if a daily oral administration of the PAMORA naldemedine as compared to placebo will reduce the risk of acute pancreatitis attacks over a 12-month period in patients with recurrent acute pancreatitis.

Secondary objectives are to compare the following parameters between naldemedine and placebo-treated groups over 12 months:

- Frequency and severity of pain attacks
- Patient-reported treatment efficacy
- Quality of life
- Gastrointestinal symptoms and bowel function
- The proportion of patients with new-onset diabetes and new-onset exocrine pancreatic insufficiency along with the progression of known pancreatic insufficiency measured as changes in the use of relevant medication
- Health care resource utilization
- Adherence to treatment and frequency of adverse events

Exploratory objectives are to compare the following parameters between the treatment groups after 12 months:

- Ductal and parenchymal pancreatic morphology evaluated by MRI
- Circulating blood markers of fibrosis and inflammation

### Trial design {8}

The study is a multicentre investigator-initiated, double-blind, placebo-controlled, parallel-group study of fixed-dose naldemedine. A group of 120 patients with recurrent acute pancreatitis will be prospectively included from outpatient clinics and randomized to receive oral naldemedine or matching placebo in a 1:1 ratio.

### Methods: participants, interventions, and outcomes

#### Study setting {9}

The study will be conducted at four referral centres for pancreatitis in Denmark and one in Sweden, located at Aalborg, Odense, Copenhagen (Bispebjerg and Hvidovre), and Stockholm. These centres have comprehensive experience in the management of patients with pancreatic diseases. Patients with recurrent acute pancreatitis will primarily be identified and included via the outpatient clinics at the abovementioned institutions. However, they may also be identified during hospital admission if the eligibility criteria are matched.

### Eligibility criteria {10}

#### Inclusion criteria

- Signed informed consent before any study-specific procedures
- Able to read and understand Danish or Swedish (depending on the study site)
- Adults aged 18 to 74 years (both inclusive)
- A diagnosis of recurrent acute pancreatitis with at least one attack of acute pancreatitis within the last 12 months and at least two attacks within 5 years (confirmed as defined by the revised Atlanta Criteria [17])
- Clinically stable at the time of inclusion defined by no objective, radiological or biochemical signs of acute pancreatitis or other diseases that require hospitalization
- The researcher finds that the participant understands what the study entails, is capable of following instructions, can attend when needed, and is expected to complete the study
- The investigator will ensure that fertile female participants have a negative pregnancy test before treatment initiation and use contraception during the study period. The following methods of contraception, if properly used, are generally considered reliable: oral contraceptives, patch contraceptives, injection contraceptives, vaginal contraceptive ring, intrauterine device, surgical sterilization (bilateral tubal ligation), vasectomized partner, double barrier (condom and pessary), or sexual abstinence. Methods of contraception will be documented in the source documents

#### Exclusion criteria

- Known hypersensitivity towards study medication
- Patients with known or suspected gastrointestinal obstruction or perforation or patients at increased risk of recurrent obstruction due to the potential for gastrointestinal perforation (e.g. peptic ulcer disease, acute colonic pseudo-obstruction, malignancy of the GI tract, Crohn’s disease)
- Previous pancreatic surgery including pancreaticoduodenectomy or other procedures involving the pancreatic head and sphincter of Oddi
- Known severe renal insufficiency (defined as estimated glomerular filtration rate < 30 ml/min)
- Female participants that are lactating
- Pre-existing severe comorbidities (evaluated by the investigator prior to inclusion)
- Attack of acute pancreatitis requiring admission within 4 weeks prior to inclusion
- Gallstone aetiology of recurrent acute pancreatitis (magnetic resonance cholangiopancreatography or endoscopic ultrasound excluding biliary stones must be available prior to enrolment)
- Treatment with strong CYP3A4-inhibitors (itraconazole, ketoconazole, ritonavir, indinavir, saquinavir, telithromycin, grapefruit juice and clarithromycin), strong CYP3A inducers (e.g. St. John’s wort (Hypericum perforatum), rifampicin, carbamazepine, phenobarbital and phenytoin) or P-glycoprotein inhibitors (e.g. cyclosporine).

#### Who will take informed consent? {26a}

Medical doctors affiliated with the study or trained site staff with relevant knowledge of the disease and conducting clinical trials will give the potential participants information regarding the study. The participant will be informed thoroughly about the purpose of the study, the specific procedures the study entails, and potential benefits and risks. All patients will be informed orally and written before deciding whether they want to participate in the study. Furthermore, they will be informed that they can withdraw from the study at any given time without giving a reason. The interview will take place at the respective inclusion sites at the outpatient clinic.

#### Additional consent provisions for collection and use of participant data and biological specimens {26b}

When the participant signs the informed consent form, they will be asked to fill in a separate consent form if they are willing to contribute their blood samples to a biobank.

### Interventions

#### Explanation for the choice of comparators {6b}

Since there is no established preventive treatment for recurrent acute pancreatitis, the comparator is a matched placebo tablet with an identical appearance to the active tablets but without the active component naldemedine.

#### Intervention description {11a}

Patients with recurrent acute pancreatitis will receive oral treatment with naldemedine 0.2 mg daily (standard clinical dose) or matched placebo tablets once daily. Naldemedine and placebo will be presented as identical tablets, taken with approximately 100 ml water. Since the aim is to evaluate the long-term effects of PAMORAs on the frequency of pancreatitis attacks and disease progression, a long treatment period of 12 months has been chosen.

#### Criteria for discontinuing or modifying allocated interventions {11b}

If a patient experiences intolerable side effects when taking 0.2 mg naldemedine or placebo daily, a single downward dose titration to one tablet every other day is allowed, with this dose being the final dosage for the remainder of the study. Typical side effects associated with naldemedine are diarrhoea, abdominal pain, and other mild gastrointestinal symptoms. These treatment-related symptoms are not associated with a higher discontinuation rate than placebo [16].

A participant should be withdrawn from trial, if at any time:

- It is the wish of the participant for any reason
- The investigator judges it necessary due to medical reasons
- The investigator judges severe non-compliance to protocol
- They experience intolerable side effects despite tapering the dose of naldemedine to 0.2 mg (or matching placebo) every other day

#### Strategies to improve adherence to interventions {11c}

A detailed count of the study medication dispersion and return will be kept for all participants. Patients will be asked to fill out a study drug diary to document adherence. Furthermore, patients will be followed by monthly telephone consultations with the study personnel during the entire study period.

#### Relevant concomitant care permitted or prohibited during the trial {11d}

Patients with recurrent acute pancreatitis included in the study will continue their scheduled visits and examinations in the outpatient clinic while participating in the study. Patients will also receive routine treatment deemed necessary by the responsible physician according to the usual standard of care and clinical guidelines. All medications are registered in the patient’s case report form (CRF) at baseline, including name, strength, frequency of dosing, and reason for use. In case of a change in medication during the trial period (dose change or discontinuation), this will be documented in the CRF. Patients will be asked to phone the study centre at any time if they need to speak to study personnel, should they require additional concomitant medication.

#### Provisions for post-trial care {30}

When patients reach the end of the study or discontinue treatment for other reasons, they will all revert to their routine visits and examinations at their respective tertiary outpatient clinics.

All participants with unresolved events (adverse event (AE) and serious adverse event) at the end of the study, except those who dropped out before randomization or starting active treatment, must be included in safety follow-up visits until the symptoms resolve or are deemed stable by the treatment-responsible doctor.

All participants will be covered by the patient insurance of the respective site of trial conduction. Participants are advised to seek consultancy from their insurance company if they plan to travel during or right after participation in the study, as participation in a trial involving medical treatment may alter private insurance status.

### Outcomes {12}

#### Primary outcome

The primary outcome is time to recurrence of acute pancreatitis verified by the revised Atlanta Criteria. This outcome is compared between the group receiving naldemedine and the placebo group during the 12-month treatment period.

The revised Atlanta Criteria is the current gold standard for the diagnosis of acute pancreatitis, and it requires a minimum of two out of three of the following features:

(i) Abdominal pain typical for acute pancreatic (acute onset of a persistent, severe, epigastric pain often radiating to the back)
(ii) Serum lipase or amylase levels at least three times greater than the upper limit of normal
(iii) Characteristic findings of pancreatic inflammation on contrast-enhanced computed tomography, MRI, or transabdominal ultrasonography [17]

#### Secondary outcomes

- Difference between treatment groups in disease severity assessed by the Atlanta Criteria
- The difference between treatment groups in frequency and severity of pain attacks (without fulfilling acute pancreatitis criteria), assessed by questionnaires and monthly interviews after the 12-month period
- Difference in patient’s global impression of change between treatment groups assessed by a questionnaire at 12 months follow-up
- Changes in quality of life between treatment groups from baseline to 12 months follow-up assessed by a questionnaire
- Changes in gastrointestinal symptoms and bowel function between treatment groups from baseline to 12 months follow-up assessed by questionnaires
- Proportion of patients with new-onset diabetes according to the WHO criteria [18] and changes in endocrine pancreas function between treatment groups from baseline to 12 months follow-up assessed by haemoglobin A1c (HbA1c)
- Proportion of newly diagnosed exocrine pancreatic insufficiency defined by the use of pancreatic enzyme replacement therapy or changes in exocrine pancreas function between treatment groups from baseline to 12 months follow-up assessed by a faecal-elastase test
- Difference between treatment groups in health care resource utilization (measured in frequency and type of health services used, e.g. admission rate and duration) during the 12-months treatment period
- Difference between treatment groups in adherence to treatment and frequency of adverse events during the 12-months treatment period assessed by study drug diary, return of unused medicine, and regular interviews

#### Explorative outcomes

- Changes in pancreatic morphology (pancreas volume/size, fibrosis, fatty infiltration, and ductal pathology) between treatment groups from baseline to 12 months follow-up measured by MRI
- Difference in circulating markers of fibrosis and inflammation from baseline to 12 months follow-up

#### Participant timeline {13}

The participant timeline is shown in Table 1.

**Table 1:**
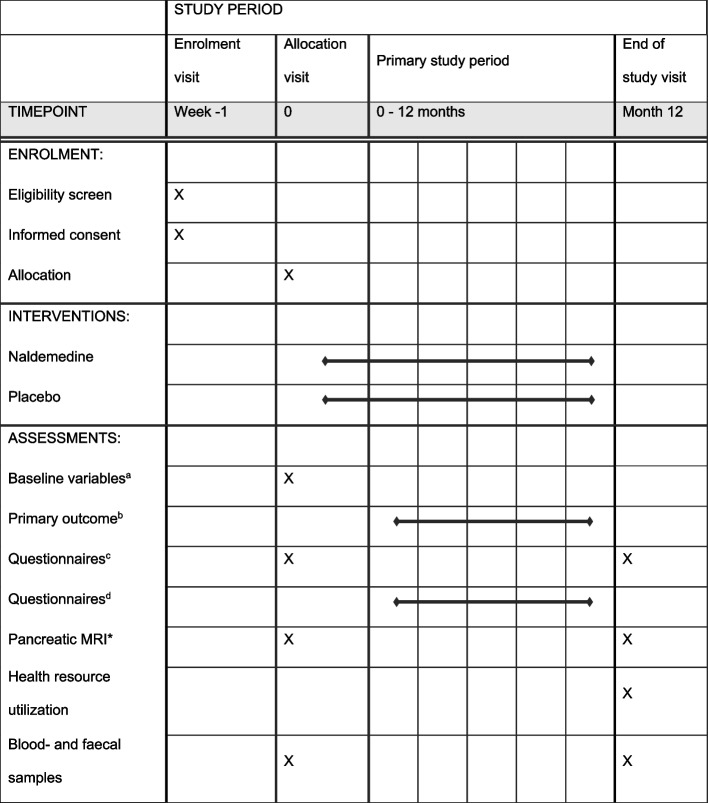
Schedule for enrolment, interventions, and assessments including visits for patients

^**a**^Baseline variables: sex; age; the number of recurrences of acute pancreatitis before inclusion, presence of chronic pancreatitis and aetiology of pancreatitis; use of opioids, weak analgesics, and other medication; alcohol consumption and smoking status; Information regarding diabetes mellitus and use of pancreatic enzyme replacement therapy; Previous invasive treatment for pancreatitis; Comorbidities based on the Charlson Comorbidity Index [19]

^**b**^Verified recurrence(s) and severity of acute pancreatitis based on the Atlanta Criteria and pain attacks not fulfilling the Atlanta Criteria; Pain attack diary and the modified Brief Pain Inventory Short Form are filled in once for each attack

^**c**^Patient Global Impression of Change questionnaire and European Organization for Research and Treatment of Cancer Quality of Life questionnaire (EORTC QLQ-C30); Gastrointestinal Symptom Rating Scale and Bristol Stool Form Scale

^**d**^Study drug diary and adverse events diary

*Magnetic resonance imaging

#### Sample size {14}

The sample size calculation is based on the following assumptions:

- Included recurrent acute pancreatitis patients will have one pancreatitis attack at least once pr. year
- Naldemedine is expected to reduce the pancreatitis attack rate by 50%
- Power is set to 90% at a 2-sided alpha level of 0.05

These assumptions analysed by the extended (recurrent) Cox model yield a required sample size of 120 patients observed for 12 months. We implement a planned interim analysis after 60 participants have been enrolled to allow re-estimation of sample size requirements and to examine adverse event rates.

#### Recruitment {15}

For patients with recurrent acute pancreatitis, recruitment material will be placed in waiting rooms at the outpatient clinics. Patients interested in participating in the study may contact study personnel for further information. Furthermore, to identify eligible patients with recurrent acute pancreatitis from the respective departments or outpatient clinics, medical history and previous treatment can be passed on from the treatment-responsible physician from medical records to study personnel. Potentially eligible patients from the outpatient clinic may be contacted by study personnel, but only if the treatment-responsible physician has granted permission. Patients can be assessed for inclusion 4 weeks after discharge from a pancreatitis attack if they are clinically stable at the time of inclusion. The expectation is to enrol five patients per week at the five inclusion sites.

### Assignment of interventions: allocation

#### Sequence generation {16a}

Randomization is performed in blocks (block-randomization) using approved statistical software. Since opioid consumption is assumed to be an important prognostic variable, patients will be stratified before randomization creating two strata based on opioid treatment. Naldemedine and matching placebos will be requisitioned and delivered by the Hospital Pharmacy at Aalborg University Hospital. All medication will be labelled with the randomization number corresponding to the allocation and clearly state that it is only intended for use in a clinical trial.

#### Concealment mechanism {16b}

The Hospital Pharmacy will create a randomization list at Aalborg University Hospital based on the sequence described above. The list is sequentially numbered, where newly included patients are assigned the lowest available number consecutively.

#### Implementation {16c}

After inclusion, the Hospital Pharmacy will assign a specific randomization number to the individual patient from a predefined randomization list. This randomization number will be returned to study personnel, blinded to the allocation alongside the participants. None of the collected data are expected to unblind the randomization.

### Assignment of interventions: blinding

#### Who will be blinded {17a}

In this double-blind study, labelling and blinding is performed by Wasdell Europe Limited, according to Annex 13 of the Good Manufacturing Practice guidelines of the European Commission, ICH, GCP guidelines. Research pharmacists perform subsequent handling and distribution of the investigational medicine at the Hospital Pharmacy at Aalborg University Hospital. This blinding mechanism ensures that personnel and participants directly involved in the project are prevented from obtaining information that might bias the results.

#### Procedure for unblinding if needed {17b}

If a participant needs urgent medical care that requires knowledge of the given treatment randomization, the treatment allocation for each participant is available at the study centres in sealed envelopes (provided by the Hospital Pharmacy at Aalborg University Hospital). They are stored in a secure area accessible to personnel involved in the study authorized by the investigator to open the code for a single subject. This procedure allows unblinding of individual subjects without revealing the codes of the entire study. Thus, the study personnel can determine which treatment a subject was given by opening the sealed envelope with the corresponding randomization number.

## Data collection and management

### Plans for assessment and collection of outcomes {18a}

#### Clinical outcome and health resource utilization

The clinical outcomes will be documented through questionnaires and a pain attack diary, filled in before, during, and after treatment. Participants will be asked to fill in the pain attack diary, and the modified Brief Pain Inventory Short Form for each pancreatitis attack during the study period. Hospital admissions and whether patients fulfilled the Atlanta criteria for acute pancreatitis during admission will subsequently be documented using the medical files along with the severity of the attack. To assess the effects of the treatment on quality of life, patients will also be asked to fill in the following: (i) the European Organization for Research and Treatment of Cancer Quality of Life Questionnaire (EORTC QLQ-C30) and (ii) the Patient Global Impression of Change Questionnaire. For a detailed description of these questionnaires, see the section below. The patients’ medical files at 12 months follow-up are used to document health resource utilization, readmission rate, and total length of hospital stays during the recovery phase of pancreatitis.

#### Pain attack diary

The number and severity of pain attacks are registered by the patient in a paper diary, documenting the duration of each pain attack and the need for medication or hospital admission. Participants will also be asked to phone study personnel in connection to each attack.

#### The modified Brief Pain Inventory Short Form

The modified Brief Pain Inventory Short Form is used to subjectively document patients’ pain if pain attacks are experienced during the treatment period. The questionnaire consists of 14 items based on a visual analogue scale from 0 to 10 documenting current pain, pain within the previous 24 h, and impact on various daily functions [20].

#### Patient Global Impression of Change

Patient Global Impression of Change is a seven-point rating scale for self-reporting a patient’s overall experienced treatment efficacy on their symptoms [21], which is filled out at the end of the study visit after 12 months.

#### The European Organization for Research and Treatment of Cancer Quality of Life

The EORTC QLQ-C30 Questionnaire is used to document life quality, physical function, and several other health-related parameters [22] and will be filled in by participants at baseline and 12 months follow-up. The questionnaire has been validated for assessing the quality of life in patients with chronic pancreatitis and is composed of single-item measures and multi-item scales by scores ranging from 0 to 100 after linear transformation of the raw score [23]. A high score on the functional scale represents a high level of functioning, as does a high score for global health status, while a high score for the symptom items represents a high level of symptomatology.

#### Bristol Stool Form Scale

The Bristol Stool Form Scale is a patient-generated assessment of stool frequency and stool consistency and aids the researcher in the assessment of intestinal function [24].

#### Gastrointestinal Symptom Rating Scale

The Gastrointestinal Symptom Rating Scale is a disease-specific instrument of 15 items combined into five clusters depicting abdominal pain, reflux, constipation, diarrhoea, and indigestion. It consists of a seven-point graded Likert-type scale, where 1 represents absence of symptoms and 7 represents very troublesome symptoms. The validity and reliability of the questionnaire are well-documented, and normal values for a general population are available [25].

#### Biochemistry

Patients will have their pancreatic exocrine and endocrine pancreatic function evaluated using faecal-elastase test and HbA1c at baseline and at 12 months follow-up. These samples are analysed immediately.

#### Imaging

If possible, included patients will undergo MRI at baseline and at 12 months follow-up to evaluate changes in pancreatic morphology. The following features will be extracted: Anatomical images for volumetric assessment of the pancreas, 3-dimensional magnetic resonance cholangiopancreatography for evaluation of the ductal system, diffusion-weighted imaging for evaluation of pancreatic fibrosis, and Dixon imaging for assessment of fatty infiltration [26, 27]. Additional potential imaging biomarkers for pancreatic fibrosis are performed for patients referred to Aalborg University Hospital, including T1 mapping for fibrosis and MR elastography for assessing pancreatic stiffness.

#### Plans to promote participant retention and complete follow-up {18b}

All patients will be followed by monthly telephone interviews with the study personnel during the entire study period and visits in the outpatient clinic at baseline and 12 months. If a participant does not turn up for a scheduled visit, every effort will be made to contact the participant. In any circumstance, every effort will be made to document the participant’s health status. In case of discontinuation or withdrawal from the study before the planned 12 months period, the patient is subsequently invited to a premature “end of study”-visit, which includes an MRI scan, blood samples, and questionnaires to ensure data completeness.

#### Data management {19}

Data related to the primary outcome, clinical outcome, questionnaires, and health resource utilization will be entered directly into electronic CRFs using REDCap (short for Research Electronic Data Capture) [28, 29], licensed by Aalborg University Hospital, and saved electronically. All forms are filled out during (or immediately after) the assessment of a subject and must be complete. Errors and corrections are logged as provided by the REDCap interface. It is possible to export validated data from REDCap to, e.g. statistical software for further analysis. When data have been entered, reviewed, and verified, the data will be frozen to prevent editing. Digitalized data are backed up and stored on specific drives at each site under the responsibility of the principal investigators for a minimum of 5 years after the study has ended.

#### Confidentiality {27}

Personal information on potential or enrolled patients will only be collected by personnel authorized and trained for the task and documented by a delegation- and training log available for the study monitors. All study personnel are subject to professional secrecy. Data on participants are stored safely in the following places:

(i) In REDCap that offers password protection, logging, and user-level control
(ii) In a locked file cabinet in a locked room at the respective study site

#### Plans for collection, laboratory evaluation, and storage of biological specimens for genetic or molecular analysis in this trial/future use {33}

Patients will have routine clinical blood sample tests performed (Prothrombin, HbA1c, glucose, electrolytes, vitamin D, amylase, albumin, creatinine, carbamide, alanine aminotransferase, alkaline phosphatase, gamma-glutamyl transferase, bilirubin, thyrotropin, parathyrin, and cholesterol) corresponding to 18.5 ml blood at baseline and at 12 months follow-up. These samples are analysed immediately. Patients will also have blood tests drawn to detect circulating markers of fibrosis and inflammation (C-reactive protein, interleukin (IL)-4, IL-6, IL-8, IL-10, IL-12, IL-18, tumour necrosis factor-alpha, transforming growth factor beta-1 (TGF-1), soluble fractalkine (s-Fr), CD163, PRO-C11 (released N-terminal pro-peptide of type XI collagen), PRO-C3 (released N-terminal pro-peptide of type III collagen), PRO-C4 (internal epitope in the 7S domain of type IV collagen), PRO-C6 (C-terminal of released C5 domain of type VI collagen α3 chain (endotrophin)) and PRO-C22 (C-terminal of type XXII collagen)) corresponding to 31 ml blood. Samples will be frozen and stored at − 80 °C in a freezer at the respective inclusion sites until analysis. After the analyses, blood samples will be kept in the biobank for future research purposes and destroyed at the latest 15 years after the termination of the study. Based on the blood samples drawn at baseline (18.5 ml + 31 ml blood) and 12 months follow-up (18.5 ml + 31 ml blood), a total of 99 ml blood will be drawn from each participant during the entire study period.

### Statistical methods

#### Statistical methods for primary and secondary outcomes {20a}

The Andersen-Gill model is applied for the statistical analyses of the primary endpoint. It is an extension of the Cox model and can be used for recurrent event analyses. This analysis enables efficient use of patient data since the same patient can contribute with multiple events in case of more than one recurring pancreatitis attack over the 12-month treatment period. The output of the analysis is a hazard ratio indicating whether naldemedine-treated patients have a lower hazard of recurring pancreatitis events compared to placebo. All analysis will be done with the intention to treat principle. Secondary endpoints, including questionnaires, biochemical parameters, and MRI assessment parameters, are analysed using regression models. Logistic regression models are used to compare dichotomous outcomes between treatment groups.

#### Interim analyses {21b}

A blinded interim analysis will be performed after 60 participants have been enrolled to assess safety regarding excessive adverse events and event rate for recurrent acute pancreatitis in the cohort to re-estimate sample size requirements.

#### Methods for additional analyses (e.g. subgroup analyses) {20b}

Covariate and subgroup analysis will be performed to investigate if there are patient-related factors that can predict the effect of the treatment. Subgroup analyses are planned for suspected important factors for treatment efficacy, such as the aetiology of recurrent acute pancreatitis and the use of opioids.

#### Methods in analysis to handle protocol non-adherence and any statistical methods to handle missing data {20c}

A per-protocol analysis of the primary endpoint will be presented to illustrate the efficacy of the treatment under ideal conditions (i.e. full compliance). The “Last observation carried forward” method is utilized in case of missing data.

#### Plans to give access to the full protocol, participant-level data and statistical code {31c}

The trial has been registered at clinicaltrials.gov and EudraCT. All results will be published as open access whenever possible using scientific and public media. After finalization, the key anonymized trial data will be accessible through public databases, e.g. Zenodo (www.zenodo.org) [30].

### Oversight and monitoring

#### Composition of the coordinating centre and trial steering committee {5d}

The project coordination will be led by the research group from the primary study centre Mech-Sense at Aalborg University Hospital, where the sponsor-investigator is located. The trial steering committee is composed of the appointed primary investigators from each of the five study sites. The steering committee will arrange monthly meetings to coordinate and streamline study activities. Ad-hoc support will be available and provided day-to-day by the responsible researchers at Mech-Sense. The central trial database is also managed by and located at Aalborg University Hospital. The trial sponsor at Aalborg University Hospital is responsible for study oversight and supervision of all study sites, and a plan for these procedures and an oversight log have been prepared.

#### Composition of the data monitoring committee, its role and reporting structure {21a}

The trial is performed in accordance with The International Council for Harmonisation guidelines for good clinical practice (GCP) as required by law for all studies involving investigational medicinal products in the European Union. Therefore, the study and all sites will be monitored by independent GCP monitors not directly involved in the study. The monitor will assure that the participant’s rights, safety, and well-being are maintained during the study in accordance with the Declaration of Helsinki. The GCP monitor will also ensure that collected data are valid, complete, and well documented.

#### Adverse event reporting and harms {22}

Any clinically significant abnormalities will be reported as an AE. The primary investigator is responsible for the assessment of clinical significance. Since the patients included in the study suffer from a severe medical condition, they are expected to develop specific symptoms and laboratory result deviations frequently associated with the disease during the study period. The symptoms and associated conditions have been presented to the regulatory authorities and pre-approved to be excluded from AE reporting.

AE's and adverse reactions will be registered until 55 h after the study medication is discontinued. This timeframe corresponds to five times the half-life of naldemedine, which is 11 h [31]. All expected or unexpected AEs and adverse reactions are registered in the CRF. They are reported in a final report which will be recorded into EudraCT along with the results at the trial's termination. Information about AEs will be collected from the first administration of any investigational product until the end of the study. The Research Ethics Committee and Medicines Agencies will annually be presented with a list of all serious adverse reactions and suspected unexpected serious adverse reactions that have occurred during the trial, along with an evaluation of patient safety until the trial has concluded. All fatal or life-threatening suspected unexpected serious adverse reactions are reported to the Health and Medicines Authorities and The Research Ethics Committees as soon as possible and no later than seven days after the sponsor has been notified of suspected unexpected serious adverse reactions.

#### Frequency and plans for auditing trial conduct {23}

An independent monitor will be allocated from the GCP unit at each study site. The responsible monitor will contact and visit the principal investigator regularly. The monitor will be authorized to inspect the different study records (electronic CRFs, source data/documents, and other relevant data), ensuring that the subjects' information is kept confidential following the data protection agency’s conditions. It will be the monitor's responsibility to inspect CRFs regularly throughout the study to ensure compliance and completion of the protocol and that consistent and accurate data is entered in these. The monitor will verify that each subject has given written informed consent for direct access to study records and study procedures. The principal investigator will cooperate with the monitor to ensure that all potential problems discovered during the monitor’s visit will be solved. Investigator provides direct access to source data/documents (including medical records) during monitoring, auditing, or inspection by the Danish Medicines Agency or The Danish Data Protection Agency and their Swedish counterparts.

#### Plans for communicating important protocol amendments to relevant parties (e.g. trial participants, ethical committees) {25}

In case of protocol amendments, both the Danish Medicines Agency and The North Denmark Region Committee on Health Research Ethics and their Swedish counterparts will be contacted. In case of minor changes, this will be in the form of a notification. A formal amendment will be devised in case of more extensive changes (i.e. changes to in- or exclusion criteria or equivalent). All protocol modifications will be communicated to all study sites and personnel immediately.

#### Dissemination plans {31a}

The trial results, irrespective of whether positive, negative, or inconclusive, will be published in peer-reviewed scientific journals and disseminated to the public. Results may also be used in submission to regulatory authorities. The first author will be appointed according to the Vancouver system. The investigator will inform the Danish Medicines Agency and the Research Ethics Committee after the termination of the trial. No later than 90 days after trial termination, the “Declaration of the end of trial form” must be submitted to The Danish Medicines Agency. As soon as possible or within one year, the results will be registered with EudraCT. The Research Ethics Committee will be notified of the results from this study. Published articles are sent to The Danish Medicines Agency and the Research Ethics Committee.

## Discussion

Recurring acute pancreatitis is associated with pain and decreased mental and physical well-being. Currently, no effective treatments are available if the triggering factors cannot be identified and removed [32]. Patients with recurrent acute pancreatitis are also at high risk of developing irreversible fibrotic changes of the pancreas leading to endo- and exocrine dysfunction, chronic abdominal pain, and have an excess risk of pancreatic cancer [33].

Patients with recurrent pancreatitis are often exposed to excess levels of exogenous and endogenous opioids that may impair pancreatic duct clearance and thus lead to premature intrapancreatic protease activation and increased risk of new pancreatitis events. PAMORAs have been identified as a novel pharmacological treatment strategy for patients with recurrent acute pancreatitis as they facilitate pancreatic duct clearance and ameliorate opioid-induced harmful effects on the gastrointestinal tract [13, 14]. In this study, the PAMORA naldemedine will be administered orally for 12 months in a multicentre, double-blind, randomized controlled trial including 120 patients with recurrent acute pancreatitis at various disease stages of pancreatitis. The treatment effects will be investigated using several endpoints, including time to pancreatitis recurrence (primary endpoint), along with several secondary endpoints focused on the patient-reported outcomes such as pain, gastrointestinal symptoms, and quality of life. Changes in endocrine and exocrine function are documented by biochemical tests and initiation of enzyme replacement therapy and glucose-lowering therapy during the trial period. Assessments of compliance and adverse events are included to document the feasibility of long-term naldemedine treatment in patients with recurrent acute pancreatitis. Exploratory outcomes, including MRI and circulating blood markers of inflammation and fibrosis, are used to document the effect of naldemedine on disease progression and to unravel associated mechanisms.

Since recurrent acute pancreatitis is a relatively rare disease with an incidence rate estimated to 5–10 cases pr. 100,000 person-years [34] every effort to maximize the catchment area has been made in this multinational study in order to secure sufficient recruitment.

In conclusion, the PAMORA-RAP study is an investigator-initiated double-blind, randomized controlled trial investigating the effects of naldemedine on pancreatitis recurrence rate and disease progression in patients with recurrent acute pancreatitis. If successful, the study will be the first to demonstrate a pharmacologic intervention for the prevention of recurrent acute pancreatitis.

## Trial status

Recruitment was initiated in January 2022 and the study is estimated to be finalized (last patient last visit) in April 2024.

## Funding {4}

The PAMORA-RAP study is an investigator-initiated trial. It is funded as part of an unrestricted grant by the Novo Nordisk Foundation. Shionogi BV also supports the study through a supply of study drug materials. The financial supporters have no influence on publication, study design, and data collection.

## Availability of data and materials {29}

The lead coordinating research group from Mech-Sense at Aalborg University hospital will have full access to the final trial dataset through the joint REDCap database. All other study sites will have full access to their respective dataset. The study funders will have no access or rights regarding the raw data generated during the study.

## Abbreviations

AE: Adverse event
CRF: Case report form
EORTC QLQ-C30: European Organization for Research and Treatment of Cancer Quality of Life questionnaire
EudraCT: European Union Drug Regulating Authorities Clinical Trials Database
GCP: Good Clinical Practice
HbA1c: Haemoglobin A1c
MRI: Magnetic resonance imaging
PAMORA: Peripherally acting µ-opioid receptor antagonist
REDCap: Research Electronic Data Capture
